# Geographic disparities and determinants of fertility outcomes in Bangladesh

**DOI:** 10.1016/j.pmedr.2026.103617

**Published:** 2026-09-06

**Authors:** S.M. Mostafa Kamal, Efehan Ulas, Yesim Senayli

**Affiliations:** a Department of Mathematics, Islamic University, Kushtia 7003, Bangladesh; b Department of Biostatistics and Medical Informatics, Kirklareli University, Kirklareli, Turkey; c Department of Anesthesiology and Reanimation, Kirklareli University,Kirklareli, Turkey

**Keywords:** Fertility, Spatial analysis, Multilevel analysis, Regression analysis, Bangladesh

## Abstract

**Objectives:**

Regional disparities in maternal and reproductive health persist in developing countries. This study investigates geographical differences and hierarchical factors influencing fertility in Bangladesh.

**Methods:**

Using 2017–18 Bangladesh Demographic and Health Survey data, this study models the cumulative fertility of ever-married women aged 15–49. It applies a two-level hierarchical multilevel Poisson regression to separate individual socioeconomic factors from community context. Additionally, global Moran's I and local Getis-Ord (Gi*) analyses identify geographical hot and cold spots of childbearing across communities.

**Results:**

Significant geographic clustering of fertility in Bangladesh, with hot spots in Sylhet and Chittagong and cold spots in the northwest and southwest. Regression indicated that individual factors like child marriage and low maternal education increased parity. Community factors such as socioeconomic disadvantage and high fertility norms also independently influenced reproductive outcomes.

**Conclusions:**

Fertility behaviors in Bangladesh are shaped by both individual traits and the normative characteristics of shared spaces. National family planning campaigns are no longer enough; interventions must focus on community-level, geographically segregated strategies that address local infrastructural and sociocultural barriers within high-fertility hot spots.

## Highlights

• Regional fertility variations exist across geographic zones in Bangladesh.
• Child marriage and low education levels significantly elevate fertility risks.
• Shared community environments shape reproductive choices beyond individual traits.
• Targeted regional family planning strategies are vital for sustainable growth.

## 1 Introduction

Bangladesh's fertility rate dropped from 6.3 in 1975 to 3.3 in 1999–2000, remaining steady through the 1990s. It declined further to 2.3 in 2011 and stabilized until 2017–18, despite limited early socioeconomic and health development.

The decline largely results from Bangladesh's successful family planning program started in the 1960s. Contraceptive use increased from 5% in 1975 to 52% in 2017–18, mainly due to dedicated workers and strategies like private-sector involvement and facility-based services (Phillips and Hossain, 2003; Das, 2016; Islam et al., 2020).

Despite achievements, Bangladesh hasn't reached replacement fertility (2.1 births per woman). Significant regional differences remain in fertility rates and contraceptive use across eight divisions. Khulna, Rajshahi, and Rangpur have reached replacement or lower fertility, while other regions have higher fertility levels.

While fertility research in Bangladesh is well-documented, most studies use macro-level divisions or basic regressions that treat communities as uniform. These methods overlook sub-divisional differences and how micro-level social environments influence reproductive behavior. This study advances previous work by linking structural geography and social norms using two-level Poisson multilevel modeling and localized spatial analysis (Getis-Ord Gi*). This approach separates individual socio-demographic factors from community influences, identifies local fertility norm diffusion, and locates geographical hot spots across administrative boundaries. Shifting focus from administrative zones to socio-spatial spaces, the study examines regional fertility variation and key individual- and community-level factors affecting the number of children ever born Mean Number of Children Ever Born (MNCEB) among women in Bangladesh with detailed, nationally representative data.

## 2 Methods

### 2.1 Study design and population

Data for this study were drawn from the 2017–18 Bangladesh Demographic and Health Survey (BDHS), a nationally representative cross-sectional survey collecting comprehensive socioeconomic, demographic, and health data from 20,127 ever-married women aged 15–49. The survey used a two-stage stratified sampling design, first selecting clusters (enumeration areas) then households. Informed written and verbal consent was obtained from all participants. The survey was conducted by the National Institute of Population Research and Training (NIPORT) under the Ministry of Health and Family Welfare, with technical assistance from ICF International and funding from United States Agency for International Development (USAID).

To evaluate the social and structural environment of reproductive behaviors, community-level variables were created by systematically aggregating individual socio-demographic data—like female education, poverty, and early marriage—based on Primary Sampling Units (PSUs). This approach converts individual traits into shared community characteristics.

To address non-normal distributions and skewness across clusters, these continuous measures were converted into dichotomous variables (‘High“ or “Low” exposures). The thresholds were set by the national cluster-level median. Dichotomizing these variables prevents model misspecification from non-linear relationships between predictors and fertility counts. From a public health perspective, median-based categories simplify interpreting odds or incidence rate ratios, providing clear benchmarks for maternal health administrators to identify and target underperforming regions.

This study used secondary data from the Bangladesh Demographic and Health Survey (BDHS), approved by the Bangladesh Medical Research Council and ICF Institutional Review Board (IRB). Participants gave written consent before data collection. Since the study used de-identified, retrospective data from the BDHS Program, no additional IRB approval or participant consent was necessary.

### 2.2 Measures

The outcome variable was fertility, measured as the mean number of children ever born MNCEB among women at the time of the survey. Individual-level explanatory variables included: current age (15–24, 25–34, 35–49 years), age at first marriage (<18 vs. ≥18 years), experience of child mortality (no vs. yes), women's education (no education, primary, secondary, higher), ideal family size (≤2 vs. ≥3 children), household wealth index (poorest, poorer, middle, richer, richest), religion (Islam vs. others), women's working status (not working vs. working), access to media (no vs. yes), and current contraceptive method use (no method, traditional, modern).

The eight administrative divisions were grouped into five regions based on geographic location: Dhaka as the Central region; Sylhet and Mymensingh as the Northeast; Rangpur and Rajshahi as the Northwest; Chittagong and Barisal as the Southeast; and Khulna as the Southwest region.

Community factors included residence (urban vs. rural) and cluster-level data like women's education, media access, fertility, and age at first marriage, categorized into “low” or “high” based on median values. The household wealth index was created by NIPORT via principal component analysis of household assets, as detailed in the BDHS report (NIPORT, 2020).

### 2.3 Statistical analysis

We used univariate analyses to summarize respondents' background characteristics and chi-square tests for bivariate associations. A two-level multilevel model was specified, with individual women (Level 1) nested within community clusters (Level 2). Regional variables were included as fixed effects, not as a third level, to avoid restricting degrees of freedom. This captures macro-level variations and isolates true community-level variance in the random intercepts. Given the hierarchical data and count outcome, we used two-level Poisson regression to identify factors related to MNCEB. We weighted the data for representativeness, with both weighted and unweighted samples shown in Table 1. Regression coefficients were exponentiated to IRRs. We set significance at 0.05 and ran analyses in Stata 15.0.

**Table 1:** Unweighted and weighted percentage distribution of the number of children ever born among ever-married women aged 15–49 years by geographic region in Bangladesh, 2017–18.⁎

Number of Children	Central	North-East	North-South	South-East	South-West	Total
	unweighted *n* / weighted *N* (%)	unweighted *n* / weighted *N* (%)	unweighted *n* / weighted *N* (%)	unweighted *n* / weighted *N* (%)	unweighted *n* / weighted *N* (%)	unweighted *n* / weighted *N* (%)
0	351 / 601 (11.7%)	432 / 274 (10.0%)	453 / 457 (8.8%)	485 / 457 (9.6%)	272 / 237 (10.1%)	1993 / 2025 (10.1%)
1	714 / 1207 (23.6%)	872 / 535 (19.6%)	1096 / 1093 (21.1%)	1019 / 924 (19.5%)	627 / 537 (23.0%)	4328 / 4296 (21.3%)
2	843 / 1442 (28.1%)	1088 / 662 (24.2%)	1631 / 1636 (31.6%)	1326 / 1187 (25.0%)	858 / 763 (32.6%)	5746 / 5690 (28.3%)
3	541 / 928 (18.1%)	816 / 513 (18.7%)	978 / 1017 (19.6%)	1006 / 970 (20.4%)	544 / 503 (21.5%)	3885 / 3931 (19.5%)
4	281 / 504 (9.8%)	513 / 325 (11.9%)	545 / 589 (11.4%)	601 / 576 (12.1%)	208 / 187 (8.0%)	2148 / 2181 (10.8%)
5	133 / 242 (4.7%)	337 / 215 (7.8%)	234 / 248 (4.8%)	308 / 307 (6.5%)	74 / 70 (3.0%)	1086 / 1081 (5.4%)
6	58 / 104 (2.0%)	168 / 109 (4.0%)	77 / 84 (1.6%)	180 / 183 (3.8%)	27 / 25 (1.1%)	510 / 505 (2.5%)
7	36 / 65 (1.3%)	96 / 58 (2.1%)	34 / 37 (0.7%)	92 / 98 (2.1%)	16 / 13 (0.6%)	274 / 271 (1.3%)
8	7 / 13 (0.3%)	41 / 27 (1.0%)	14 / 14 (0.3%)	29 / 32 (0.7%)	3 / 2 (0.1%)	94 / 88 (0.4%)
9	7 / 12 (0.2%)	24 / 15 (0.5%)	2 / 3 (0.0%)	8 / 8 (0.2%)	1 / 1 (0.0%)	42 / 39 (0.2%)
10	3 / 6 (0.1%)	5 / 4 (0.1%)	0 / 0 (0.0%)	3 / 3 (0.1%)	0 / 0 (0.0%)	11 / 12 (0.1%)
11	0 / 0 (0.0%)	4 / 2 (0.1%)	3 / 3 (0.1%)	1 / 1 (0.0%)	0 / 0 (0.0%)	8 / 5 (0.0%)
12	0 / 0 (0.0%)	0 / 0 (0.0%)	0 / 0 (0.0%)	1 / 1 (0.0%)	0 / 0 (0.0%)	1 / 1 (0.0%)
13	0 / 0 (0.0%)	0 / 0 (0.0%)	1 / 1 (0.0%)	0 / 0 (0.0%)	0 / 0 (0.0%)	1 / 1 (0.0%)
Total	2974 / 5123 (100%)	4396 / 2738 (100%)	5068 / 5182 (100%)	5059 / 4747 (100%)	2630 / 2336 (100%)	20,127 / 20,127 (100%)

⁎ n represents unweighted sample sizes; N represents weighted sample sizes; % represents weighted percentages indicates the weighted percentage distribution within each respective region.

In the models, the response variable is the MNCEB, representing the number of children for each woman in a region. MNCEB was chosen over metrics like the Total Fertility Rate (TFR) or Parity Progression Ratios (PPR). TFR shows macro trends but lacks the individual variation needed for detailed modeling, while PPR focuses on specific birth transitions. MNCEB offers a comprehensive measure of lifetime reproductive output and reflects socio-geographical influences. To address limitations such as age-related parity and cohort effects, we included women's age as a control variable in all models. Adjusting for age ensures that spatial and multilevel findings reflect true socio-environmental factors, not age biases.

To keep the empirical focus concise, the textbook derivations for the two-level Poisson framework are omitted, and the model is specified directly. Let Y_ij denote the cumulative fertility count for individual i in community cluster j. Given the random intercept u_j, the count follows a Poisson distribution with a log-linear link defined as:

ln(μ_ij) = β_0 + β X_ij + γ Z_j + u_j.where X_ij represents the vector of individual-level socio-demographic predictors, Z_j represents the vector of community-level and fixed regional contextual factors, and u_j represents the unobserved between-cluster random variance, assumed to be normally distributed.

We used robust statistical and econometric software for all computations. We used two-level random-intercept multilevel models with poisson in Stata (Version 17), with Maximum Likelihood estimation and Adaptive Gauss-Hermite Quadrature for precision. I computed spatial autocorrelation (Moran's I) and hot-spot mapping (Getis-Ord Gi*) in ArcGIS Pro (Version 3.0), using a row-standardized spatial weight matrix based on fixed distance thresholds to model neighborhood-level effects.

To generate unbiased, nationally representative estimates from the complex survey, the framework incorporates sampling weights, clustering, and stratification. Standard weights can bias variance in hierarchical setups, so we used two-level weight scaling. Weights were adjusted at individual (Level 1) and cluster (Level 2) levels, using Stata's standard procedures, aligning selection probabilities with the true population without inflating the sample size.

The survey's clustering effect is captured by the Level-2 random intercept (u_j), which models residual correlation among women in the same community. Stratification was included via indicators—urban/rural and administrative divisions—as fixed effects. Adjusting for weights, cluster effects, and stratification, the model controls for selection and spatial design effects, ensuring valid nationwide inferences.

Unlike standard least squares, R-squared isn't valid for evaluating explained variance in hierarchical generalized linear models for counts. Instead, variance explained at Level 2 was calculated using Snijders and Bosker's proportional reduction in variance, comparing the null model's intercept variance with that of the fully adjusted Poisson model(6). This shows the reduction in between-cluster heterogeneity from adding covariates. Clearly describing this process ensures replicability and aligns with standards.

A regression diagnostic verified the validity and integrity of the findings. Model fit was checked with log-likelihood ratio tests, Akaike Information Criterion (AIC), and Bayesian Information Criterion (BIC), confirming improved fit with multi-level predictors. VIF values stayed below 5.0, avoiding severe multicollinearity. Residuals confirmed the model's stability. Monitoring of influential points showed no undue impact, ensuring robustness of the demographic transition coefficients.

Given that the dependent variable MNCEB is a non-negative count, a Poisson regression was used. A formal overdispersion test showed minor overdispersion, mainly due to large sample size. To ensure valid inferences, Poisson Quasi-Maximum Likelihood Estimation (QMLE) with robust standard errors was employed, producing consistent, unbiased estimates even with overdispersion. The Poisson model also provided better convergence and stability with complex spatial clusters and random effects. Sensitivity analyses confirmed similar results against a Negative Binomial model, validating our primary choice.

Econometric theory demonstrates that the robust Poisson estimator yields consistent and unbiased parameter coefficients even when overdispersion is present, bypassing the fragile distributional constraints of the Negative Binomial model. Furthermore, the Poisson specification offered superior computational convergence and numerical stability when integrating complex simultaneous spatial cluster operations and two-level random effects. Sensitivity analyses comparing the robust Poisson structure against a Negative Binomial specification yielded virtually identical coefficients and significance thresholds, validating the robustness of the primary model choice.

Spatial autocorrelation assessed cluster proximity; positive autocorrelation shows similar value clustering. Hot spot analysis with Getis-Ord Gi* identified significant hot and cold spots of children ever born. Analyses used ArcGIS 10.4.1.

To ensure clarity and transparency with complex survey weights, the manuscript distinguishes between raw sample configurations and weighted population estimates. Frequencies for all cross-tabulations and univariate distributions are reported as unweighted sample sizes (n) to indicate actual respondent counts. Conversely, associated proportions, means, and percentages are calculated and reported as weighted estimates (%). This separation meets demographic reporting standards for large-scale surveys, adjusting for unequal selection probabilities across strata while maintaining transparency of the physical sample size.

## 3 Results

### 3.1 Background characteristics of women

Table 2 shows women by background and region. Younger women were more common in the Central and South-East. Child marriage was highest in the North-East and lowest in the North-West. Child mortality was highest in the North-East and lowest in the South-West. Higher education was most common in the Central region and least in the North-East. Preference for small families (≤2 children) was highest in the South-West. Rural residence, poverty, working status, and media access varied by region, with contraceptive use lowest in the South-East.

**Table 2:** Percentage distribution of ever-married women aged 15–49 years by demographic and socioeconomic background characteristics across geographic regions in Bangladesh, 2017–18.

Background characteristics							
	Central	North-East	North-West	South-East	South-West	Total	χ^2^ *P*-value
Current age							*P* < 0.01
15–24	29.4	28.2	26.3	29.2	25.1	27.9	
25–34	36.9	35.1	34.2	34.5	33.4	35.0	
35–49	33.7	36.6	39.4	36.2	41.5	37.1	
Type of marriage							*P* < 0.01
Adult	28.7	31.4	16.7	27.4	19.8	24.7	
Child	71.3	68.6	83.3	72.6	80.2	75.3	
Child mortality							*P* < 0.01
No	86.7	81.2	83.4	84.4	87.3	84.6	
Yes	13.3	18.8	16.6	15.6	12.7	15.4	
Level of education							*P* < 0.01
No education	16.3	20.4	20.1	12.3	13.6	16.6	
Primary	30.0	37.5	30.6	30.5	29.7	31.2	
Secondary	39.5	32.3	37.4	44.2	43.9	39.6	
Higher	14.2	9.8	11.9	13.0	12.7	12.6	
Ideal no. of children							*P* < 0.01
<3	79.7	70.1	81.6	69.0	86.0	77.1	
≥3	19.7	28.9	17.9	30.3	13.3	22.2	
Non-numeric	0.6	1.0	0.5	0.7	0.7	0.7	
Residence							*P* < 0.01
Urban	51.6	17.1	17.5	25.0	22.5	28.5	
Rural	48.4	82.9	82.5	75.0	77.5	71.5	
Household wealth							*P* < 0.01
Poorest	8.1	27.1	27.9	18.0	12.3	18.6	
Poorer	12.4	23.7	25.3	17.8	22.2	19.7	
Middle	16.2	17.6	21.1	22.6	25.1	20.2	
Richer	28.4	17.1	15.7	19.3	22.8	20.8	
Richest	34.9	14.5	10.1	22.3	17.7	20.8	
Religion							*P* < 0.01
Islam	93.4	90.2	89.5	91.8	85.5	90.7	
Others	6.6	9.8	10.5	8.2	14.5	9.3	
Currently working							*P* < 0.01
No	64.1	53.8	36.5	62.9	38.1	52.3	
Yes	35.9	46.2	63.5	37.1	61.9	47.7	
Access to media							*P* < 0.01
No	22.0	48.3	36.2	38.7	29.3	34.0	
Yes	78.0	51.7	63.8	61.3	70.7	66.0	
Current contraceptive use							*P* < 0.01
Not using	41.0	44.1	36.8	47.6	39.0	41.7	
Traditional method	9.0	8.6	9.6	8.8	11.8	9.4	
Modern method	50.0	47.3	53.7	43.5	49.2	49.0	
Total no. of women	5123	2738	5182	4747	2336	20,127	

Table 3 presents the mean number of children ever born MNCEB by background characteristics and region. The national average MNCEB was 2.38 ± 1.64. Significant associations (*p* < 0.01) were observed between MNCEB and most background characteristics across regions, except for religion. The null model showed significant between-cluster variation in MNCEB (ICC = 0.0067, *p* < 0.01). Adding individual- and community-level variables reduced the variance, with Model III explaining about 93.88%. Decreases in AIC confirmed better fit.

**Table 3:** Mean number and standard deviation of children ever born to ever-married women aged 15–49 years by demographic and socioeconomic background characteristics across geographic regions in Bangladesh, 2017–18.

Background characteristics	Region						χ^2^ *P*-value
	Central	North-East	North-West	South-East	South-West	Total	
Current age							*P* < 0.01
15–24	0.90 ± 0.77	1.15 ± 0.91	0.98 ± 0.91	1.02 ± 0.82	0.89 ± 0.74	0.99 ± 0.81	
25–34	2.17 ± 1.12	2.58 ± 1.40	2.21 ± 1.40	2.47 ± 1.20	2.06 ± 0.95	2.31 ± 1.16	
35–49	3.46 ± 1.64	4.02 ± 1.93	3.23 ± 1.93	3.89 ± 1.70	2.92 ± 1.29	3.52 ± 1.66	
Type of marriage							*P* < 0.01
Adult	1.69 ± 1.30	2.19 ± 1.77	1.60 ± 1.12	1.93 ± 1.43	1.49 ± 1.18	1.82 ± 1.42	
Child	2.42 ± 1.66	2.92 ± 1.91	2.43 ± 1.48	2.80 ± 1.81	2.27 ± 1.32	2.57 ± 1.67	
Child mortality							*P* < 0.01
No	1.93 ± 1.36	2.25 ± 1.62	1.97 ± 1.24	2.20 ± 1.51	1.88 ± 1.15	2.05 ± 1.40	
Yes	4.11 ± 1.79	4.51 ± 1.89	3.92 ± 1.43	4.51 ± 1.71	3.77 ± 1.34	4.21 ± 1.70	
Level of education							*P* < 0.01
No education	3.46 ± 1.89	3.83 ± 2.15	3.30 ± 1.54	4.00 ± 2.00	3.03 ± 1.42	3.55 ± 1.86	
Primary	2.60 ± 1.60	2.88 ± 1.83	2.67 ± 1.42	3.11 ± 1.77	2.62 ± 1.31	2.80 ± 1.64	
Secondary	1.75 ± 1.21	2.08 ± 1.46	1.79 ± 1.11	2.09 ± 1.38	1.76 ± 1.08	1.89 ± 1.27	
Higher	1.27 ± 0.98	1.26 ± 1.02	1.23 ± 0.94	1.40 ± 1.11	1.18 ± 0.99	1.28 ± 1.01	
Ideal no. of children							*P* < 0.01
<3	1.93 ± 1.39	2.22 ± 1.64	2.08 ± 1.32	2.15 ± 1.46	1.98 ± 1.25	2.07 ± 1.42	
≥3	3.37 ± 1.82	2.26 ± 2.06	3.19 ± 1.63	3.53 ± 1.49	3.04 ± 1.46	3.42 ± 1.86	
Non-numeric	3.32 ± 2.93	3.61 ± 2.34	4.08 ± 2.50	3.18 ± 2.53	3.50 ± 1.87	3.73 ± 2.53	
Residence							*P* < 0.01
Urban	2.02 ± 1.44	2.08 ± 1.62	2.00 ± 1.25	2.44 ± 1.66	2.08 ± 1.34	2.11 ± 1.46	
Rural	2.45 ± 1.74	2.71 ± 1.90	2.36 ± 1.50	2.60 ± 1.78	2.14 ± 1.33	2.49 ± 1.70	
Household wealth							*P* < 0.01
Poorest	2.79 ± 1.69	2.90 ± 2.00	2.57 ± 1.56	2.92 ± 1.92	2.29 ± 1.34	2.74 ± 1.78	
Poorer	2.74 ± 1.79	2.77 ± 1.90	2.35 ± 1.47	2.86 ± 1.88	2.28 ± 1.37	2.61 ± 1.72	
Middle	2.50 ± 1.80	2.63 ± 1.89	2.20 ± 1.39	2.52 ± 1.68	2.15 ± 1.39	2.40 ± 1.65	
Richer	2.07 ± 1.60	2.53 ± 1.82	2.10 ± 1.43	2.44 ± 1.65	2.05 ± 1.30	2.21 ± 1.59	
Richest	1.91 ± 1.28	2.29 ± 1.64	1.87 ± 1.17	2.14 ± 1.52	1.85 ± 1.19	1.99 ± 1.37	
Religion							*P* = 0.11
Islam	2.21 ± 1.61	2.73 ± 1.92	2.29 ± 1.48	2.61 ± 1.78	2.16 ± 1.36	2.40 ± 1.67	
Others	2.26 ± 1.36	2.27 ± 1.56	2.25 ± 1.31	2.03 ± 1.25	1.94 ± 1.14	2.15 ± 1.32	
Currently working							*P* < 0.01
No	2.07 ± 1.52	2.39 ± 1.87	1.88 ± 1.40	2.30 ± 1.66	1.89 ± 1.45	2.14 ± 1.60	
Yes	2.47 ± 1.69	3.04 ± 1.87	2.53 ± 1.44	2.99 ± 1.81	2.27 ± 1.30	2.65 ± 1.65	
Access to media							*P* < 0.01
No	2.83 ± 1.82	3.01 ± 2.06	2.69 ± 1.61	2.94 ± 1.92	2.42 ± 1.39	2.83 ± 1.84	
Yes	2.05 ± 1.49	2.34 ± 1.64	2.06 ± 1.31	2.30 ± 1.58	2.00 ± 1.29	2.14 ± 1.47	
Current contraceptive use							*P* < 0.01
Not using any method	1.95 ± 1.77	2.40 ± 2.06	2.02 ± 1.66	2.30 ± 1.88	1.93 ± 1.50	2.13 ± 1.81	
Traditional method	2.69 ± 1.52	3.56 ± 1.81	2.79 ± 1.55	3.34 ± 1.94	2.51 ± 1.25	2.96 ± 1.68	
Modern method	2.34 ± 1.42	2.81 ± 1.68	2.39 ± 1.24	2.69 ± 1.50	2.18 ± 1.18	2.48 ± 1.43	
Total	2.22 ± 1.60	2.69 ± 1.90	2.29 ± 1.46	2.56 ± 1.75	2.12 ± 1.33	2.38 ± 1.64	

### 3.2 Regional variations in fertility

Significant regional differences persisted in the fully adjusted models. Compared with the Central region, MNCEB remained significantly higher in the North-East and South-East regions and lower in the North-West and South-West regions. The final model shows child marriage linked to higher fertility. Education protects, with educated women having lower MNCEB. Larger desired families, lower wealth, no media, and rural living also increase fertility. Community media access wasn't significantly linked, as its confidence interval crossed unity, indicating no independent effect. Living in high-fertility or early marriage communities raised MNCEB.

### 3.3 Results of spatial analysis

**Fig. 1:**
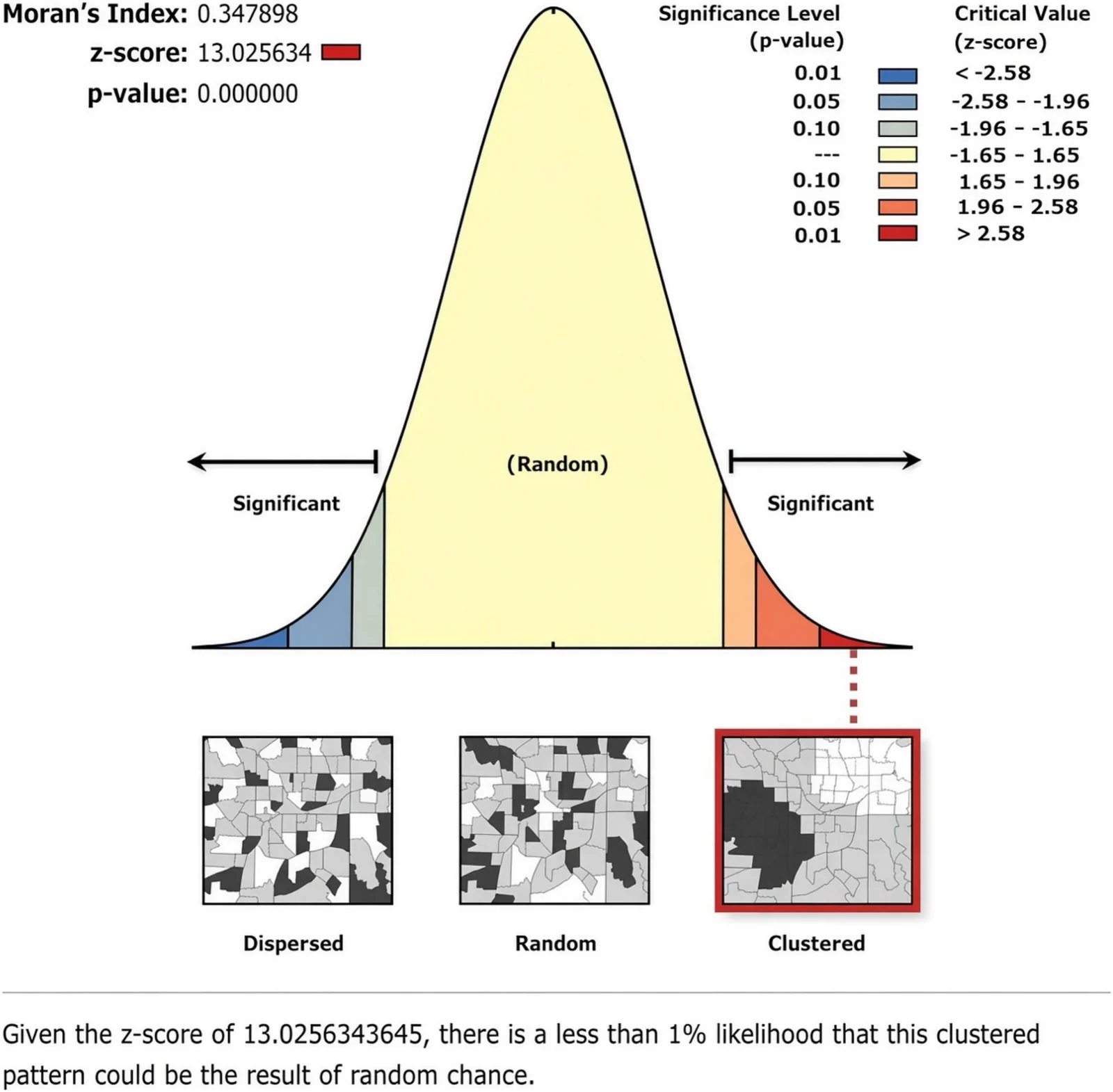
Global Moran's Index analysis of spatial autocorrelation for the number of children ever born among ever-married women aged 15–49 years by geographic region in Bangladesh, 2017–18: delineating a statistically significant, non-random spatial clustering pattern.

**Fig. 2:**
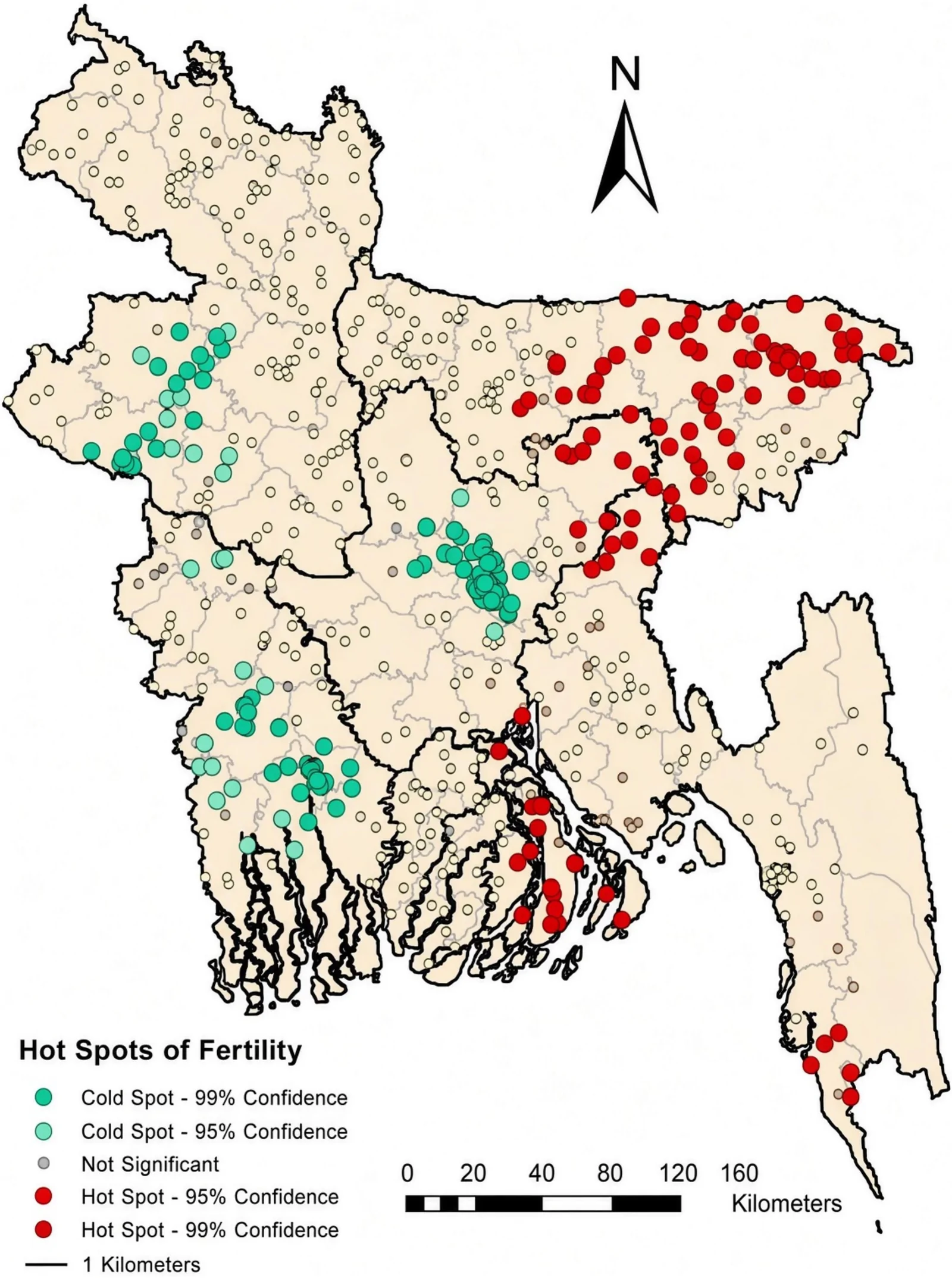
Hot spot and cold spot analysis (Getis-Ord (Gi*)) of spatial clustering for fertility among ever-married women aged 15–49 years across districts in Bangladesh, 2017–18: delineating localized reproductive risks by exact 90%, 95%, and 99% statistical confidence thresholds.

Global Moran's I (0.348, *p* < 0.01) confirmed significant spatial clustering of fertility (Fig. 1). Hot spot analysis identified the North-East and South-East as areas of significantly higher fertility, whereas the North-West and South-West (and parts of the Central region) were cold spots (Fig. 2). These spatial findings were consistent with the multilevel regression results (Table 4). A sensitivity analysis re-estimated the multilevel Poisson regression by including all eight Bangladesh divisions as fixed effects, using Dhaka as the baseline, to validate regional classification boundaries and reduce potential bias.

**Table 4:** Incidence rate ratios and 95% confidence intervals from multilevel Poisson regression models for individual- and community-level determinants of the number of children ever born among ever-married women aged 15–49 years in Bangladesh, 2017–18.

Explanatory variables	Null Model	Model I	Model II	Model III
Individual- and socioeconomic level variables				
Current age (*ref*: (Mason, 1989)–24)				
25–34			2.08 (2.02–2.15)^a^	2.09 (2.03–2.16)^a^
35–49			2.77 (2.68–2.87)^a^	2.78 (2.69–2.88)^a^
Type of marriage (*ref*: adult)				
Child			1.21 (1.18–1.24)^a^	1.22 (1.19–1.25)^a^
Child mortality (*ref*: no)				
Yes			1.47 (1.44–1.50)	1.45 (1.42–1.49)
Level of education (*ref*: no education)				
Primary			0.98 (0.96–1.01)	0.98 (0.96–1.01)
Secondary			0.90 (0.87–0.92)^a^	0.90 (0.87–0.92)^a^
Higher			0.72 (0.69–0.75)^a^	0.73 (0.70–0.76)^a^
Ideal no. of children (*ref*: <3)				
≥3			1.25 (1.22–1.27)^a^	1.22 (1.20–1.25)^a^
Non-numeric			1.38 (1.26–1.50)^a^	1.33 (1.23–1.45)^a^
Household wealth (*ref*: poorest)				
Poorer			0.99 (0.96–1.02)	0.99 (0.96–1.02)
Middle			0.98 (0.95–1.01)	0.99 (0.95–1.01)
Richer			0.98 (0.95–1.01)	0.99 (0.96–1.02)
Richest			0.95 (0.91–0.98)^b^	0.97 (0.94–1.00)^b^
Religion (*ref*: Islam)				
Others			0.91 (0.88–0.94)^a^	0.92 (0.89–0.95)^a^
Currently working (*ref*: no)				
Yes			1.01 (0.99–1.03)	1.01 (0.99–1.03)
Access to media (*ref*: no)				
Yes			0.94 (0.92–0.96)^a^	0.95 (0.93–0.97)^a^
Current contraceptive use (*ref*: not using any method)				
Traditional method			1.17 (1.14–1.21)^a^	1.18 (1.14–1.21)^a^
Modern method			1.20 (1.18–1.23)^a^	1.21 (1.18–1.23)^a^
Community-level variables				
Region (*ref*: central)				
North-East		1.19 (1.14–1.25)^a^	1.08 (1.05–1.12)^a^	1.04 (1.00–1.06)^b^
North-West		1.03 (0.99–1.08)	0.93 (0.90–0.96)^a^	0.94 (0.91–0.97)^a^
South-East		1.14 (1.09–1.19)^a^	1.06 (1.03–1.09)^a^	1.03 (1.00–1.06)^b^
South-West		0.95 (0.90–1.00)^b^	0.90 (0.87–0.97)^a^	0.93 (0.90–0.97)^a^
Residence (*ref*: urban)				
Rural				1.02 (1.00–1.05)^b^
Women's education (*ref*: low)				
High				1.00 (0.98–1.03)
Access to media (*ref*: low)				
High				0.96 (0.91–1.01)^a^
Women's fertility (*ref*: low)				
High				1.17 (1.14–1.19)^a^
Age at marriage (*ref*: low)				
High				0.95 (0.92–0.98)^a^
Community-level variance (Standart Error(SE))	0.02228 (0.020)	0.0158258 (0.002)	0.00351 (0.001)	0.00121 (0.001)
Interclass Corellation Coefficient = Variance Partition Coefficient	0.0067	0.0016	0.0011	0.0004
Explained variation (%)	Ref.	76.11	0.8358	93.88
Akaike Information Criterion	73,514.31	73,391.29	72,738.63	60,354.35
-Log likelihood	36,755.15	36,689.65	36,359.31	30,149.17

**Note:** a indicates statistical significance at the p < 0.01 level; b indicates statistical significance at the *p* < 0.05 level. Values in parentheses represent 95% confidence intervals.

The findings from this separate division model demonstrated exceptional empirical robustness. The estimated incidence rate ratios, standard errors, and statistical significance levels for all key individual-level attributes (e.g., maternal education, age at first marriage) and community-level contextual variables remained highly stable and unaltered. Furthermore, the separate-division specification confirmed that the reproductive risks were significantly and disproportionately elevated in the Sylhet and Chittagong divisions, which aligns perfectly with the historical East-West demographic gradient documented in Bangladeshi fertility literature. This consistency proves that our spatial and multilevel inferences are fully independent of regional aggregation boundaries.

## 4 Discussion

This study highlights significant regional differences in fertility across Bangladesh, with higher rates in the North-East and South-East, and lower in the North-West and South-West, even after accounting for individual and community factors. Spatial analysis identified these areas as hot and cold spots, indicating geographic influence beyond personal traits, with a Global Moran's I of 0.348 (*p* < 0.01). These disparities, persistent over two decades, reflect structural inequalities, with the North-East and South-East lagging in female education, child marriage, and contraceptive use, while the North-West and South-West show better socioeconomic conditions and more advanced fertility transition. The findings extend previous research, illustrating uneven family planning success across Bangladesh.

Our adjusted multilevel framework shows community media access isn't statistically significant, with confidence intervals crossing one. This suggests that just having mass media available at the community level doesn't lead to reproductive behavioral changes. Instead, fertility transitions depend more on individual active media consumption and personal socio-demographic factors than passive neighborhood media exposure.

This clustering shows reproductive behavior in Bangladesh depends on individual traits and shared space's features. Our spatial analysis reveals community context—like local marriage customs and fertility norms—independently influence women's fertility. This explains persistent disparities in the North-East and South-East despite national campaigns. Recognizing these micro-hotspots is crucial for targeted family planning and empowerment efforts, as a one-size-fits-all approach fails.

Elevated fertility in Bangladesh's northeastern and southeastern corridors, especially in Sylhet and Chittagong, results from deeply rooted sociocultural factors beyond socioeconomic deficits. Highly conservative religious and traditional lineage norms promote natural fertility and patriarchy, rewarding early childbearing and high parity, which determine women's social status. Norms enforce restrictions on female autonomy, with reproductive choices controlled by husbands and elder women. This social pressure, combined with fear of sanctions, fosters resistance to family planning, enabling persistent high-fertility behaviors in micro-communities.

These disparities highlight bottlenecks in Bangladesh's health system. The centralized family planning program distributes resources evenly, ignoring regional challenges. Difficult terrains like Sylhet's haors and Chittagong's hills hinder access. Hot spots with high fertility face institutional issues, such as high vacancy rates among female Family Welfare Assistant (FWA)s and clinic shortages, causing missed home visits and contraceptive stock-outs. Policies should shift from uniform centralization to decentralize resources, addressing infrastructure and geographic gaps.

Evaluating multi-level random parameters is crucial. Although the estimated Intra-class Correlation Coefficient (ICC) values are small, indicating that community cluster variance accounts for a minor part of the total variation in fertility, this aligns with international demographic models where individual biological, marital, and socioeconomic factors primarily influence childbearing. Importantly, this small contextual variance remains statistically significant across all models, showing neighborhood clustering is a real structural factor, not a sampling artifact. Even a small ICC suggests the socio-spatial environment consistently influences reproductive behaviors. For public health officials in Bangladesh, these micro-clusters are key intervention points; neglecting them means ignoring persistent structural forces trapping women in high-fertility norms despite national efforts.

Several factors strongly predicted higher MNCEB at the individual level. Women married before 18 had 22% higher fertility (Incidence Rate Ratio (IRR) = 1.22, 95% Confidence Interval (CI), 1.19–1.25), supporting evidence that child marriage increases fertility through prolonged reproductive exposure (Nahar and Zahangir, 2026; Snijders and Bosker, 2012; Kamal, 2021; Nasrullah et al., 2014; Onagoruwa and Wodon, 2018; Yaya et al., 2019). Child mortality experience was also linked to higher fertility, reflecting replacement behavior in high-mortality areas (Hossain et al., 2007; Saksena et al., 1984). The study confirms that child marriage, child mortality, lower education, larger ideal family size, poorer household wealth, and lack of media exposure are significantly associated with higher fertility. These findings align with research from Bangladesh and other developing countries (Nahar and Zahangir, 2026; Kamal, 2021; Yaya et al., 2019; Götmark and Andersson, 2020). Community factors also matter: women in clusters with high fertility norms and early marriage had higher fertility, indicating influence from shared social environments and pressures.

Higher education significantly reduced women's fertility, with a 27% lower rate (IRR = 0.73, 95% CI, 0.70–0.76) among educated women. Education boosts autonomy, contraceptive knowledge, and preference for fewer children, aligning with existing research (Nahar and Zahangir, 2026; Saksena et al., 1984; Götmark and Andersson, 2020; Rosenzweig and Schultz, 1989). Conversely, larger ideal family size, lower wealth, lack of media access, and rural dwelling were linked to higher fertility, supporting previous studies (Nahar and Zahangir, 2026,Mason, 1989).

Women in high-fertility communities and those with lower median age at first marriage showed higher MNCEB. These effects highlight social norms and reproductive behavior diffusion within clusters (Snijders and Bosker, 2012,Kamal, 2021), beyond individual traits. The North-East and South-East regions, with the highest fertility, have lower education, higher child marriage, limited media access, and lower contraceptive use. These disadvantages mirror patterns from twenty years ago (Islam et al., 2020), showing slow progress in these regions (Nahar and Zahangir, 2026, Howlader et al., 2022).

A cautious interpretation is necessary when analyzing the link between contraceptive use and fertility. The positive correlation doesn't mean family planning causes higher birth counts; it reflects reverse causality due to the survey's cross-sectional design. In Bangladesh, modern contraceptives are mainly used for fertility limitation, not spacing. Women in high-fertility areas adopt contraception after reaching desired family size, creating a paradox where users have more children than non-users. This requires targeted, parity-specific public health strategies.

Our multilevel models couldn't address key fertility determinants due to data limits and retrospective design constraints. Biological factors like breastfeeding duration, abortion history, and couples' fertility status, which influence the biological maximum of childbearing, weren't modeled. Important socioeconomic factors, such as spousal education and migration patterns, were also omitted. Although our models accounted for spatial clustering and confounding variance, future longitudinal or calendar studies are recommended to include these biological and social variables, enabling a complete analysis of reproductive transitions in Bangladesh.

Finally, the empirical utility of this study is limited by the temporal ambiguity of the cross-sectional data, as socio-demographic exposures, contraceptive behaviors, and fertility counts were recorded simultaneously. This makes establishing a clear chronological sequence difficult. Notably, estimates of contraceptive use may be affected by reverse causality, as current choices often adapt to past reproductive outcomes rather than before them.

The indicators rely on retrospective self-reported questionnaires, which may introduce recall bias, especially among older maternal cohorts regarding birth intervals or pregnancy timelines. Although robust multilevel and spatial controls are used, residual confounding may persist from unmeasured variables like breastfeeding duration, abortion history, subfecundity, and migration patterns. Using ecological aggregation across PSUs to construct community predictors helps identify norms but risks masking small within-cluster differences. These limitations are common in large surveys, but future research with longitudinal panels or calendar data is recommended to better isolate behavioral changes in Bangladesh.

## 5 Conclusion

This study reveals a significant, non-random geographical clustering of fertility behaviors across Bangladesh, showing persistent reproductive variations beyond traditional zones. Multilevel and spatial analyses strongly indicate that lifetime childbearing is systematically linked to individual socio-demographic factors and local environmental pressures. Specifically, lower female education, socioeconomic status, and child marriage are strongly associated with higher cumulative parity. Additionally, living in disadvantaged or high-fertility communities is independently linked to greater reproductive output, reflecting localized normative pressures and behavioral diffusion at the neighborhood level.

Because these empirical findings are based on statistical associations from a cross-sectional framework, macro-level policy implications should be approached with caution. While centralized national reproductive health campaigns correlate with broad declines, local cluster boundaries indicate that a uniform strategy is inadequate for sub-divisional variations. Public health efforts might benefit from targeted, community-level interventions that consider socio-spatial factors in high-fertility zones. To validate these regional fertility determinants and distinguish true causal pathways from temporary behavioral changes, future demographic research should focus on prospective longitudinal studies and multi-year cohort panels. Tracking households over time is essential to understand how changing environments influence reproductive transitions in Bangladesh.

## Declaration of generative AI and AI-assisted technologies in the manuscript preparation process

Statement: During the preparation of this work, the author(s) used Gemini and Grok to prepare text, tables, and figures to clarify the context; also, the statistical evaluations were checked in this way as a third and a fourth opinion. After using this tool/service, the author(s) reviewed and edited the content as needed and take(s) full responsibility for the content of the published article.

## CRediT authorship contribution statement

**S.M. Mostafa Kamal:** Writing – review & editing, Writing – original draft, Validation, Supervision, Software, Methodology, Formal analysis, Data curation, Conceptualization. **Efehan Ulas:** Writing – review & editing, Writing – original draft, Resources, Methodology, Investigation, Formal analysis, Data curation, Conceptualization. **Yesim Senayli:** Writing – review & editing, Writing – original draft, Visualization, Methodology, Investigation.

## Declaration of competing interest

The authors declare that they have no known competing financial interests or personal relationships that could have appeared to influence the work reported in this paper.

## Data Availability

Data will be made available on request.
